# Improvement in health-related quality of life with Botulinum toxin A injection in acquired superior oblique palsy

**DOI:** 10.3389/fmed.2023.1198380

**Published:** 2023-06-29

**Authors:** Chonglin Chen, Meiping Xu, Huanyun Yu, Yipao Li, Xinping Yu

**Affiliations:** ^1^State Key Laboratory of Ophthalmology, Zhongshan Ophthalmic Center, Sun Yat-sen University, Guangdong Provincial Key Laboratory of Ophthalmology and Visual Science, Guangzhou, China; ^2^National Clinical Research Center for Ocular Diseases, Eye Hospital, Wenzhou Medical University, Wenzhou, China

**Keywords:** acquired superior oblique palsy, Botulinum toxin A, healthy related quality of life, AS-20 questionnaire, ocular deviation

## Abstract

**Purpose:**

This study aimed to investigate the outcomes of Botulinum toxin A (BTA) injection into the inferior oblique (IO) muscle for the management of unilateral acute acquired superior oblique palsy (SOP) and to evaluate changes in health-related quality of life post-injection using the Adult Strabismus-20 (AS-20) questionnaire.

**Methods:**

A prospective cohort study was performed in patients with unilateral acute acquired SOP who received BTA injections. Four units of BTA were injected into the ipsilateral IO muscle. Ocular examinations were performed pre-and post-injection, including alignment, ocular movement, and cyclotorsion deviation. The patients’ AS-20 questionnaire scores were analyzed.

**Results:**

A total of 21 patients with acute acquired SOP were included. The initial median vertical deviation was 5 PD (range 1–16), which was improved to 0 PD (range 0–10) at both 1 and 6 months post-injection (*p* < 0.001 and *p* < 0.001, respectively). The median torsional deviation was 7° (range 2–18) at baseline and resolved to 0 degrees (range −3–5) at the 1-month and 0° (range −2–7) at the 6-month follow-up (*p* < 0.001 and *p* < 0.001, respectively). There were significant increases in the overall score (OAS), psychosocial subscale score (PSS), and functional subscale score (FSS) from baseline values at both the 1-month (*p* < 0.001, *p* < 0.001, and *p* = 0.001, respectively) and 6-month follow-up (all *p* < 0.001).

**Conclusion:**

Injecting BTA into the ipsilateral IO muscle successfully resolved vertical and torsional deviations and significantly improved quality-of-life scores. Our findings show that BTA treatment, as an early treatment for acute acquired SOP, can help patients by significantly improving their quality of life.

## Introduction

Superior oblique palsy (SOP) is the most frequently occurring form of paralytic vertical strabismus and cranial nerve palsy, and it is usually caused by trauma, vascular insufficiency, tumor or idiopathic, and congenital or iatrogenic factors (1). A diagnosis of SOP can be confirmed by clinical examination of ocular misalignment in the nine cardinal gazes, with a positive Park-Bielschowsky 3-step test (2). The superior oblique muscle has a unique anatomic function and excyclotorsional, depressing, and abducting effects on the globe (3). As a result, patients with acquired SOP presenting with intolerable vertical diplopia, torsional diplopia, or significant torticollis are indicated for treatment.

Various surgical and non-surgical treatment options are available to reduce the vertical ocular misalignment that causes diplopia or anomalous head position in acquired SOP patients. Some people have been treated with surgery after at least 6 months of observation or conservative treatment (4). Prism is one of the treatments that may be sufficient to help improve symptoms in those with mild vertical deviation (5). While acquired SOP presents with vertical diplopia, torsional diplopia, or torticollis, the effect of the prism is limited. Botulinum toxin A (BTA), the most potent type of biological toxin, blocks the release of acetylcholine at the neuromuscular junctions of cholinergic nerves, leading to muscle paralysis, and is approved for therapeutic use in different etiologies of strabismus including both acute and chronic forms (6–10), and it has been reported to be used in the treatment of SOP (11).

Acute acquired SOP can cause symptomatic diplopia, usually with anomalous head posture, which can make normal activities difficult, affect the quality of life, and cause psychological distress (12). The Adult Strabismus-20 (AS-20) questionnaire was designed to evaluate health-related quality of life and functional vision in patients with strabismus (13–15). In the present study, we evaluated the changes in quality of life, including psychosocial and functional domains, following BTA injection into the ipsilateral inferior oblique (IO) muscle for the early management of acute acquired SOP in patients using the AS-20, particularly since no such study has been reported before.

## Participants and methods

In this prospective case series, 21 patients with unilateral acute SOP who were referred to the Eye Hospital of Wenzhou Medical University from April 2020 to June 2021 were enrolled. The diagnosis of SOP was confirmed by the Park–Bielschowsky 3-step test. All patients were diagnosed and treated with BTA injections within 3 months after the onset of the condition. Patients who had undergone previous strabismus surgery, concomitant orbital fractures, other cranial nerve palsies, or contraindications (e.g., pregnancy, eye infection, and myasthenia gravis) were excluded. This study conformed to the tenets of the Declaration of Helsinki. Informed consent was obtained from all patients, and this study was approved by the Ethics Committee of the Eye Hospital of Wenzhou Medical University.

The injections were administered as follows. Topical anesthesia was achieved using tetracaine hydrochloride 1%. The conjunctiva was opened approximately 2 mm, and the IO muscle was exposed using a squint hook. Next, 4 units (100 units dissolved in 2 mL normal saline; 0.08 mL = 4 units) of BTA (Botulinum Toxin Type A for Injection, Hengli, Lanzhou, China) were injected directly into the IO muscle using a 1 mL needle. The injections were administered by a single surgeon.

Ocular examinations were performed pre-injection and 1-month and six-month post-injection. To gauge the effect of the treatment, deviation was measured in the primary position, with prisms placed in front of the paretic eye, while fixating with the healthy eye. A synoptophore exam was also conducted.

The AS-20 (Chinese version) is a strabismus-specific questionnaire that consists of 20 items divided into two domains. The “psychosocial” domain is used to measure psychosocial functioning and self-awareness, whereas the “functional” domain is used to measure physical and emotional functioning (16). The overall score (OAS) is the mean of the scores of all the answered questions. The patients completed the AS-20 questionnaire before injection (baseline) and 1 month and 6 months post-injection.

All statistical analyses were performed using SPSS (SPSS Inc., Chicago, IL, United States). The Friedman test and the Wilcoxon signed-rank test were used to analyze the changes in deviation (degrees). Changes in the AS-20 scores (before vs. after treatment) were assessed using the Wilcoxon signed-rank test. *p*-values of <0.05 were considered statistically significant.

## Results

### Demographic and clinical characteristics of patients with acquired SOP

This study included 21 patients with unilateral acute acquired SOP. The average age at diagnosis was 55.3 ± 16.5 years (range 17–75), and 76.2% (16/21) of the patients were men. In nine cases, the condition was caused by trauma, and the remaining 12 cases were caused by non-traumatic factors. In 14 patients (66.7%), the right eye was involved. All 21 patients presented with binocular diplopia in primary positions and returned a positive Parks–Bielschowsky 3-step test result. The duration of the SOP was 1.3 ± 0.6 months (range 0.5–2; Table 1).

**Table 1:** Demographic characteristics of the included patients (*n* = 21).

Characteristics	Number
**Age (years)**	
Mean ± SD	55.3 ± 16.5
Range	Range 17 to 75
**Gender**	
Male patients: female patients (numbers)	16:5
**Time since onset**	
Mean ± SD (months)	1.3 ± 0.6
Range (months)	Range 0.5 to 2.0
**Etiology (numbers)**	
Traumatic	9
Non-traumatic	12

### Treatment with BTA injection significantly resolved vertical deviation and diplopia in SOP patients in the short and long term

We recorded the deviation at baseline and at 1 month (short term) and 6 months (long term) post-injection. In all enrolled patients, the initial median vertical deviation was 5 PD (range 1–16), which improved to 0 PD (range 0–10) at both 1 month and 6 months post-injection. In addition, the median degree of torsion was 7 degrees (range 2–18) at baseline, which resolved to 0 degrees (range −3–5) at 1 month and to 0 degrees (range − 2–7) at 6 months post-injection (all *p* < 0.001; Table 2).

**Table 2:** Ocular deviation at baseline, 1 month, and 6 months after BTA injection into the inferior oblique muscle in patients with unilateral superior oblique palsy (n = 21).

	Baseline	1 month after the injection	6 months after the injection	*P* ^*^	*P_1_^#^*	*P_2_^#^*	*P_3_^#^*
	Median (range)	Median (range)	Median (range)				
**Hypertropia (PD)**							
Total	5 (1 to 16)	0 (0 to 10)	0 (0 to 10)	0.000	0.000	0.000	0.892
Traumatic	8 (2 to 14)	0 (0 to 7)	0 (0 to 10)	0.003	0.011	0.012	0.461
Non-traumatic	4 (1 to 16)	0 (0 to 10)	0 (0 to 1)	0.000	0.002	0.002	0.317
**Torsion (degrees)**							
Total	7 (2 to 18)	0 (−3 to 5)	0 (−2 to 7)	0.000	0.000	0.000	0.391
Traumatic	7 (0 to 18)	0 (0 to 5)	0 (−2 to 7)	0.000	0.008	0.008	0.783
Non-traumatic	6.5 (2 to 10)	0 (−3 to 0)	0 (0 to 0)	0.000	0.002	0.002	0.180

* Friedman test; #Wilcoxon signed-rank test. P_1_, baseline vs. 1 month; P_2_, baseline vs. 6 months; P_3_, 1 vs. 6 months; PD, prism diopters.

A subgroup analysis showed that there was a significant improvement in vertical deviation and degree of torsion in the traumatic group (*p* = 0.003 and *p* < 0.001, respectively) and the non-traumatic group (all *p* < 0.001) during the follow-up period (Table 2). No significant difference was found between the traumatic and non-traumatic groups in vertical deviation (*p* = 0.70) or torsion degree (*p* = 0.79).

At baseline, all SOP patients complained of diplopia in near and distance vision. At the 1-month follow-up, only one patient reported diplopia. At the 6-month follow-up, three patients reported diplopia, and all three patients were in the traumatic group.

### Psychosocial and functional gains after BTA injection

We recorded the patients’ responses to the AS-20 questionnaire at baseline and at 1 month and 6 months post-injection. An overall score (OAS), psychosocial subscale score (PSS), and functional subscale score (FSS) were then calculated using the patients’ responses. At both the 1- and 6-month follow-ups, there were significant increases in all the scores (OAS, PSS, and FSS) compared to the baseline values. For the OAS, compared with the baseline value (48.9 ± 11.6), significant gains were found at 1 month (82.9 ± 12.2) and 6 months (88.8 ± 14.9) post-injection (*p* < 0.001 and p < 0.001, respectively). FSS scores were found to have significantly improved compared to baseline (24.88 ± 7.4) at 1 month (80.8 ± 15.1) and 6 months (87.4 ± 19.2) post-injection (*p* = 0.001 and *p* < 0.001, respectively). PSS values also differed significantly from baseline (72.9 ± 17.1) at 1 month (85.0 ± 12.3) and 6 months (90.12 ± 11.6) post-injection (all *p* < 0.001; Table 3).

**Table 3:** Adult Strabismus 20 (AS-20) questionnaire scores at baseline and 6 months after the injection in the included patients (*n* = 21).

AS-20	Baseline		1 month		6 months		*P_1_*	*P_2_*	*P_3_*
	Mean ± SD	Median (range)	Mean ± SD	Median (range)	Mean ± SD	Median (range)			
PSSs	72.9 ± 17.1	75 (27.5 to 90)	85.0 ± 12.3	90 (57.5 to 95)	90.12 ± 11.6	95(50 to 97.5)	0.000	0.000	0.047
FSSs	24.88 ± 7.4	27.5 (10 to 40)	80.8 ± 15.1	85 (47.5 to 97.5)	87.4 ± 19.2	95 (25 to 100)	0.001	0.000	0.051
OASs	48.9 ± 11.6	52.5 (18.8 to 65)	82.9 ± 12.2	88.8 (52.5to 93.8)	88.8 ± 14.9	95 (46.25 to 97.5)	0.000	0.000	0.050

PSS, Psychosocial subscale score; FSS, functional subscale score; OAS, overall score. Wilcoxon signed-rank test. P_1_, baseline vs. 1 month; P_2_, baseline vs. 6 months; P_3_, 1 vs. 6 months; PD, prism diopters.

**Figure 1:**
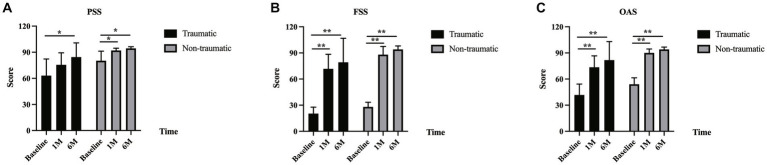
AS-20 scores in traumatic and non-traumatic groups. **(A)** There was an improvement in the psychosocial subscale score (PSS) in the traumatic and non-traumatic groups from baseline to 1-month and 6-month follow-up. The PSS in the traumatic group was 63.1 ± 19.2 at baseline, 75.6 ± 13.8 at 1 month (*p* = 0.066), and 84.4 ± 16.3 at 6 months (*p* = 0.038). The PSS in the non-traumatic group was 80.2 ± 11.1 at baseline, 92.1 ± 2.6 at 1 month (*p* = 0.003), and 94.4 ± 1.9 at 6 months (*p* = 0.002). **(B)** The functional subscale score (FSS) in the traumatic and non-traumatic groups increased from baseline to follow-up. The FSS in the traumatic group was 20.3 ± 7.4 at baseline, 71.7 ± 16.7 at 1 month (*p* = 0.008), and 79.2 ± 27.6 at 6 months (*p* = 0.008). The FSS in the non-traumatic group was 28.3 ± 5.4 at baseline, 87.7 ± 9.4 at 1 month (*p* = 0.002), and 93.6 ± 4.1 at 6 months (*p* = 0.002). **(C)** There was also an improvement in the overall score (OAS) in the traumatic and non-traumatic groups from baseline to follow-up. The OAS in the traumatic group was 41.7 ± 12.5 at baseline, 73.6 ± 13.1 at 1 month (*p* = 0.008), and 81.8 ± 21.3 at 6 months (*p* = 0.008). The OAS in the non-traumatic group was 54.3 ± 7.4 at baseline, 89.9 ± 4.5 at 1 month (*p* = 0.002), and 94.0 ± 2.7 at 6 months (*p* = 0.002).

A subgroup analysis demonstrated that there was a significant difference in the OAS in the traumatic group from baseline (41.7 ± 12.5) to 1 month (73.6 ± 13.1) and 6 months (81.8 ± 21.3) values (*p* = 0.008 and *p* = 0.008, respectively). There was an improvement in PSS in the traumatic group from baseline (63.1 ± 19.2) to 1 month (75.6 ± 13.8) and 6 months (84.4 ± 16.3) scores, and the change was not significant between baseline and 1 month (*p* = 0.066); however, the change between the baseline and 6 months was significant (*p* = 0.038). The FSS in the traumatic group was 20.3 ± 7.4 at baseline, which improved to 71.7 ± 16.7 at 1 month and to 79.2 ± 27.6 at 6 months (*p* = 0.008 and *p* = 0.008, respectively). In the non-traumatic group, the OAS, PSS, and FSS values were all significantly different when the 1-month and baseline values were compared (OAS: 54.3 ± 7.4 vs. 89.9 ± 4.5, *p* = 0.002; PSS: 80.2 ± 11.1 vs. 92.1 ± 2.6, *p* = 0.003; and FSS: 28.3 ± 5.4 vs. 87.7 ± 9.4, *p* = 0.002, respectively). There were also significant differences between the OAS, PSS, and FSS values recorded at 6 months and baseline (OAS: 54.3 ± 7.4 vs. 94.0 ± 2.7, *p* = 0.002; PSS: 80.2 ± 11.1 vs. 94.4 ± 1.9, *p* = 0.002; and FSS: 28.3 ± 5.4 vs. 93.6 ± 4.1, *p* = 0.002, respectively; Figure 1). No significant difference was found between the traumatic and non-traumatic groups in the OAS, PSS, and FSS at the 6-month follow-up.

## Discussion

Superior oblique paresis is considered the most common cyclovertical muscle palsy (17). It can be acquired or result from congenital factors. Acquired SOP is reported to be most frequently caused by trauma, and other causes of acquired cases include inflammation, infection, vascular factors, tumor, or neurological surgery (18). In this study, 9 of the cases were caused by trauma, and 12 were caused by non-traumatic factors. In the non-traumatic group, three of the cases were due to ischemia, one was due to an intracranial mass, and the other eight were idiopathic.

There are several treatment strategies for acquired SOP. Although a few studies have reported success using prisms to alleviate the symptoms of vertical diplopia, the primary treatment is surgery (5, 11). Traditionally, it is recommended to observe the patient for at least 6 months until the deviation stabilizes or heals on its own and then determine the most appropriate surgical treatment according to the situation. In our study, there was a significant improvement in the vertical deviation (*p* < 0.001) and torsion degree (*p* < 0.001), and these good outcomes are similar to those reported by Bagheri et al. (11) and Mohammad et al. (18).

We have shown that injecting BTA into the IO muscle can rapidly and safely resolve deviation in patients with acute SOP. The rate of complete recovery for SOP without treatment reportedly ranges from 50% to 65.7% at the 6-month follow-up (19, 20). Here, we achieved complete resolution of diplopia in 85.7% (18/21) of the patients at the final follow-up. The three patients who continued to experience diplopia had conditions caused by trauma. Mohammad et al. (18) achieved a favorable outcome in terms of diplopia resolution in 77% (10/12) of traumatic SOP cases, which exceeded the 66.7% (6/9) achieved in the traumatic group in our study. This discrepancy may be due to the BTA dose and injection method used. Mohammad et al. (18) injected 10–20 units of BTA (Dysport, Ipsen, Biopharm Ltd., Wrexham, United Kingdom) through the conjunctiva, whereas we injected 4 units of BTA directly into the IO muscle. It is also worth noting that the source of BTA was different between the two studies and that the active ingredients in BTA formulations produced by different manufacturers may vary. Although the active ingredients in BTA produced by different factories may differ, we suggest that the most appropriate dose and method need to be further investigated. In addition, another study reported a low recovery rate in patients with trauma-induced SOP: only three of nine such cases made a complete recovery (21). Nevertheless, there is a need to examine and determine the optimal dose and method for treating SOP with BTA.

The most significant finding of this study is that BTA injection considerably improves the quality of life. Our results showed that there was a significant improvement in the PSS, FSS, and OAS values at 1 month (*p* < 0.001, *p* < 0.001, and *p* = 0.001, respectively) and 6 months (*p* < 0.001, *p* < 0.001, and *p* < 0.001, respectively). The AS-20 score of 88.8 ± 14.9 (median 95, range 46.25–97.5) recorded post-injection compares favorably with results from other studies. In their cohort study, Hatt et al. (22) analyzed 73 consecutive adults undergoing strabismus surgery and found an average postoperative score of 78 at 6 weeks. Glasman et al. (23) demonstrated that strabismus surgery resulted in a significant improvement in quality-of-life scores, recording a median AS-20 score of 73.1 after a mean follow-up period of 91 days.

There are some limitations to this study. First, as our center is a tertiary referral hospital for eye diseases, referral bias may have been a factor. Second, despite the relatively large sample size, SOP is a comparatively rare disease, and the number of patients is limited. Third, some SOP patients can self-heal without treatment; thus, a comparative study should be conducted in the future to analyze the outcomes resulting from the natural course of this disease and those resulting from treatment.

In conclusion, injecting BTA into the IO muscle led to reduced deviation and faster recovery of function (based on quality-of-life scores) in acute acquired SOP patients. To the best of our knowledge, this is the first study to assess gains in quality of life, including the psychosocial and functional domains, experienced by patients with acquired SOP following BTA injection. Our findings suggest that BTA treatment is an early treatment for patients with acute acquired SOP with the aim of improving quality of life.

## Data availability statement

The original contributions presented in the study are included in the article/supplementary material, further inquiries can be directed to the corresponding author.

## Ethics statement

The studies involving human participants were reviewed and approved by the School of Ophthalmology and Optometry and Eye Hospital of Wenzhou Medical University, Wenzhou, Zhejiang, China. Written informed consent to participate in this study was provided by the participants’ legal guardian/next of kin.

## Author contributions

XY and HY: conceptualization. MX and YL: methodology and resources. CC: writing—original draft preparation. YL and XY: writing—reviewing and editing. All authors contributed to the article and approved the submitted version.

## Conflict of interest

The authors declare that the research was conducted in the absence of any commercial or financial relationships that could be construed as a potential conflict of interest.

## Publisher’s note

All claims expressed in this article are solely those of the authors and do not necessarily represent those of their affiliated organizations, or those of the publisher, the editors and the reviewers. Any product that may be evaluated in this article, or claim that may be made by its manufacturer, is not guaranteed or endorsed by the publisher.
